# Cancer-Associated *PIK3R1* Genetic Aberrations and Precision Medicine

**DOI:** 10.7150/ijms.109506

**Published:** 2025-06-12

**Authors:** Huan Xie, Yirong Li, Xinran Li

**Affiliations:** 1 Department of Laboratory Medicine, Zhongnan Hospital of Wuhan University, Wuhan, Hubei, China.; 2 Hubei Provincial Clinical Research Center for Molecular Diagnostics, Wuhan, China.

**Keywords:** *PIK3R1*, copy number variation, mutation, epigenetic

## Abstract

The *PIK3R1* gene encodes the class IA PI3K regulatory subunit p85α, which is frequently altered in cancer. *PIK3R1* functions as a tumor suppressor by stabilizing and inhibiting the catalytic activity of p110, and it directly binds to and enhances the activity of the PTEN lipid phosphatase. Aberrations in the *PIK3R1* gene are associated with poor prognosis in cancer; available data underscore the significant role of *PIK3R1* mutations in mediating tumorigenesis by promoting the signaling of the PI3K/AKT/mTOR pathway. Moreover, copy number variations, driver mutations, and epigenetic alterations in *PIK3R1* contribute to tumorigenesis and progression through distinct mechanisms. This article reviews the cancer-promoting effects of *PIK3R1* gene aberrations across major cancer types and elucidates their underlying mechanisms. It also discusses the targeted therapies for related aberrations, aiming to provide a comprehensive understanding of the dynamic interplay of *PIK3R1* in cancer, thereby advancing precision medicine and the development of targeted interventions.

## Introduction

The phosphoinositide 3-kinase (PI3K) signaling pathway plays a crucial role in metabolic control, immune responses, angiogenesis, and cardiovascular homeostasis, and it is among the most frequently dysregulated pathways in cancer 1-3. All PI3K catalytic subunits have a PI3K core structure consisting of a C2 domain, a helical domain, and a kinase domain 1. PI3K is categorized into three distinct classes primarily based on the presence of additional protein domains and their interaction with regulatory subunits 4. Class IA PI3K comprises a p110 catalytic subunit and a regulatory subunit, either p85 or p55. The genes *PIK3CA*, *PIK3CB*, and *PIK3CD* encode the p110α, p110β, and p110δ catalytic subunits, respectively. Meanwhile, the *PIK3R1*, *PIK3R2*, and *PIK3R3* genes encode the p85α, p85β, and p55γ regulatory subunits, respectively 5.

The p85α protein is predominantly recognized as a regulatory subunit of class IA PI3K. The *PIK3R1* gene encodes p85α, which stabilizes and inhibits the catalytic activity of p110α, while the latter catalyzes the conversion of phosphatidylinositol 4,5-bisphosphate to phosphatidylinositol 3,4,5-triphosphate (PIP3) 6. As a second messenger, PIP3 binds to a variety of target proteins within cells, thereby regulating cell proliferation, differentiation, apoptosis, metabolism, and other physiological processes 7. Additionally, the p85α regulatory protein binds directly to and enhances the activity of the PTEN lipid phosphatase, which counteracts PI3K signaling by dephosphorylating the PI3K lipid product 8, 9.

This article examines the copy number variation (CNV) of *PIK3R1*, with particular emphasis on its gene expression levels and the mechanisms through which gene dosage sensitivity influences tumorigenesis. Furthermore, the article explores the cancer-promoting effects of mutations in various domains of *PIK3R1*. We also discuss the role of epigenetic alterations in *PIK3R1* in cancer initiation and progression. Finally, treatment and management guidelines will be provided, addressing three key aspects: *PIK3R1* copy number variation, mutations in its domains, and epigenetic changes.

## Copy Number Variation of *PIK3R1* in Cancer

**Figure 1:**
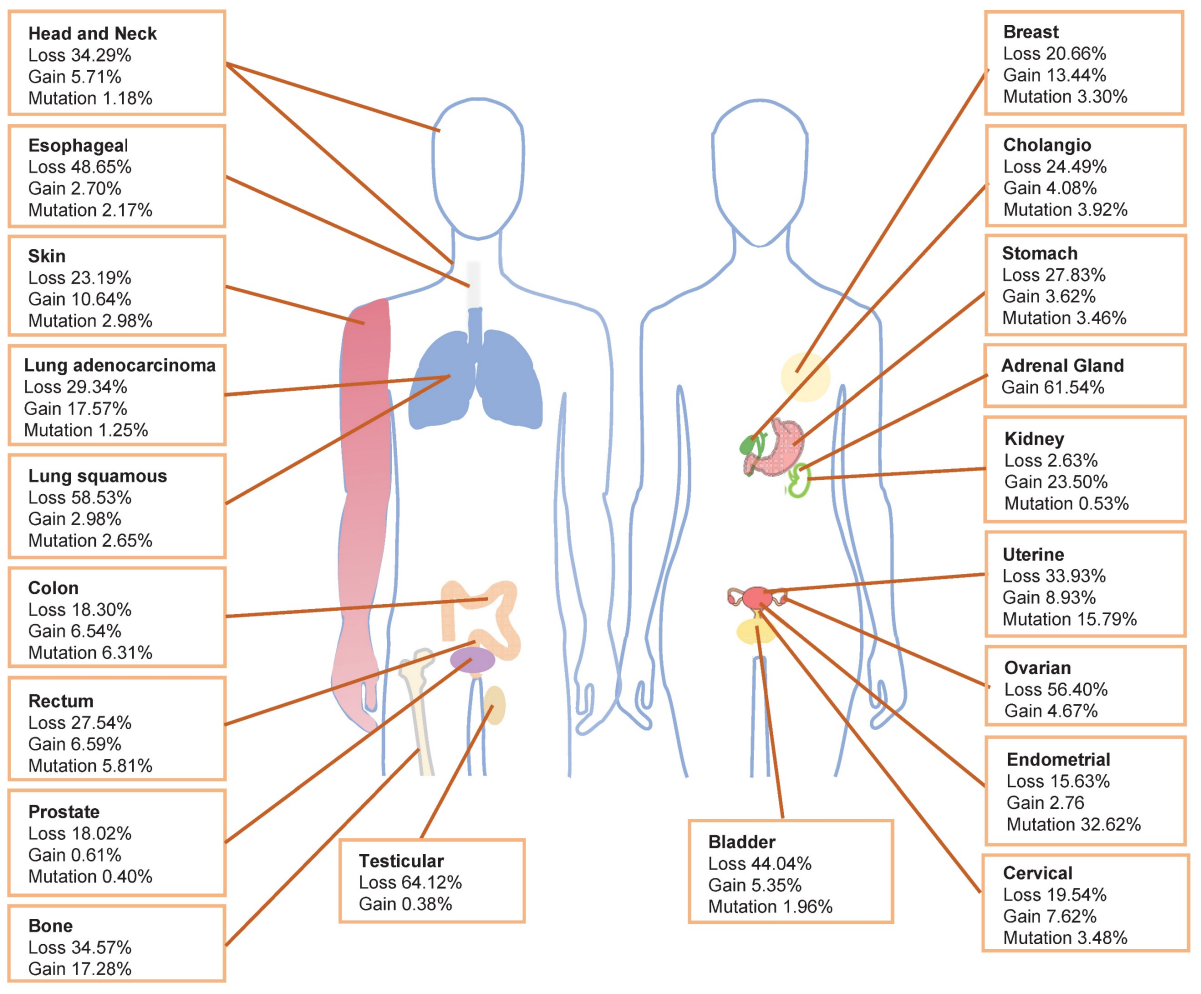
** Frequency of *PIK3R1* copy number variations and mutations.** The distribution of *PIK3R1* copy number loss, copy number gain, and mutations across various tumor types in the TCGA dataset is illustrated.

Within The Cancer Genome Atlas (TCGA) database, *PIK3R1* aberration is one of the most prevalent alterations 10. The TCGA database shows that the loss of *PIK3R1* copy number frequently occurs in various cancer types, which is consistent with the tumor suppressor role of p85α (**Fig. 1**). Cancers exhibiting *PIK3R1* copy number loss may promote tumor development through distinct mechanisms that activate downstream signaling pathways. Analysis of data from the TCGA database reveals that *PIK3R1* is lowly expressed in most tumors, compared to their corresponding normal tissues, including ovarian, prostate, breast, lung, liver, and kidney cancers 11-13.

Hemizygous deletion of *PIK3R1* is a prevalent occurrence in breast cancer, correlating with markedly reduced *PIK3R1* expression in breast tumors 14. Lower levels of *PIK3R1* expression are linked to poorer survival outcomes in breast cancer patients and contribute to tumorigenic transformations in breast cancer models. Furthermore, reduced p85α levels lead to heightened classical AKT signaling, which plays a significant role in these tumorigenic phenotypes 12, 14, 15. Research indicates that the knockdown of *PIK3R1* in breast cells triggers malignant transformation 16. In the context of prostate cancer, *PIK3R1* depletion enhances AKT phosphorylation and promotes the proliferation of prostate cancer cells 17, 18. Similarly, in renal cancer cells, the depletion of *PIK3R1* promotes AKT phosphorylation, proliferation, migration, epithelial-to-mesenchymal transition, and the emergence of stem cell-like properties via the AKT/GSK3β/CTNNB1 pathway, potentially contributing to the progression and metastasis of renal cell carcinoma 19. Among various cancers, copy-number deletion of *PIK3R1* is most commonly observed in ovarian cancer, where its deletion activates AKT and induces p110-independent JAK2/STAT3 signaling through phosphorylation changes in the docking protein Gab2. Additional mechanisms that lead to AKT activation include increased p110α kinase activity and decreased PTEN levels 20.

Haploinsufficiency of *PIK3R1* activates the PI3K pathway; conversely, homozygous deletion inhibits this pathway 21. Partial deletion of p85α enhances the p110α-p85α heterodimer's binding affinity to active receptors, thus amplifying PI3K signaling and oncogenic transformation 14, 22. On the other hand, homozygous deletion of p85α significantly reduces the amount of p110α-p85α dimers, leading to a marked decrease in PI3K activity and a reduction in PI3K-mediated biological processes such as anti-apoptosis 21.

Studies conducted in genetically engineered mouse models demonstrate that single-copy deletion of *PIK3R1* activates AKT and promotes tumorigenesis 14, 22, 23. Similar findings were reported in a mouse model of hepatocellular carcinoma characterized by liver-specific *PIK3R1* deficiency, which resulted in enhanced tumor development 13. In animal models of cancer, liver-specific knockout of *PIK3R1* has also been shown to increase PI3K pathway activation, thereby facilitating tumorigenesis 13. Additionally, experiments mimicking human tumors revealed that knockdown of p85α resulted in p110α conjugation to p85β, increased MAP4 interactions, enhanced integration with endosomal membranes, and augmented interactions with activated receptors, culminating in intensified agonist-stimulated PI3K/AKT signaling 24. Furthermore, low *PIK3R1* expression correlates with poor prognosis across multiple cancer types 11. The reduction of *PIK3R1* is particularly associated with dismal prognoses in breast and lung cancers, potentially due to its critical role in stabilizing PTEN 3, 12, 25. Thus, *PIK3R1* deletion activates downstream AKT signaling and facilitates tumorigenesis through various mechanisms across different cancer types, which is associated with unfavorable prognoses for patients.

Notably, *PIK3R1* copy-number gain remains under-discussed in the literature. Amplification of *PIK3R1* is present in 0.04% of cases recorded in the AACR GENIE database 26. Additionally, in the TCGA database, breast invasive ductal carcinoma, lung adenocarcinoma, clear cell renal cell carcinoma, adenocarcinoma of unknown primary origin, and adrenal cortex carcinoma have the greatest prevalence of *PIK3R1* copy number ampilfication **(Fig. 1)**.

## Mutation Landscape of *PIK3R1* in Cancer

**Figure 2:**
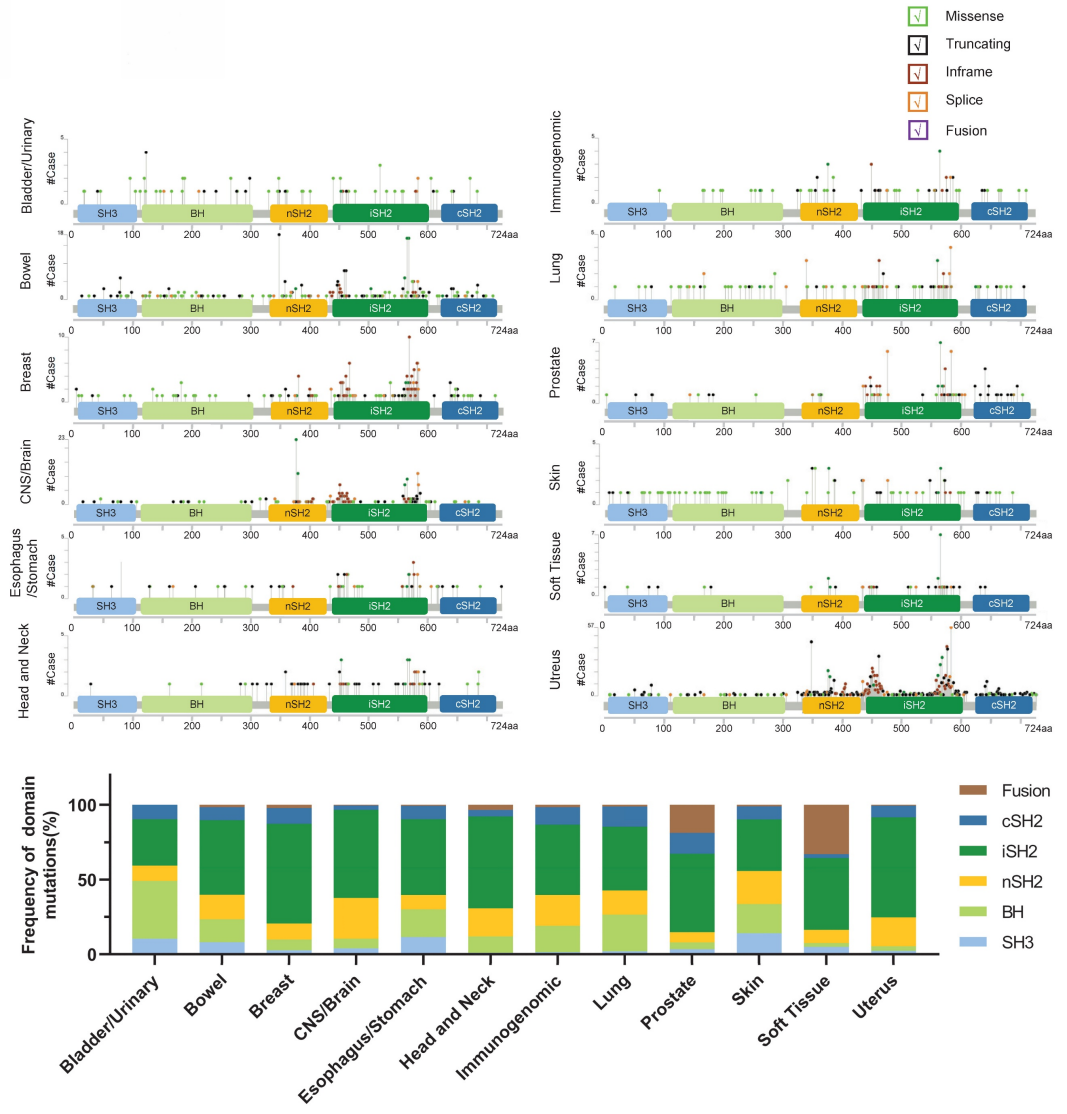
** Schematic representation of the *PIK3R1* protein and its mutation frequency.** The *PIK3R1* gene encodes the p85 regulatory subunit, which comprises 724 amino acids and five structural domains: the SH3 domain, the BH domain, the nSH2 domain, the iSH2 domain, and the cSH2 domain. (Upper) The mutation frequency of *PIK3R1* across different tissues is shown, based on data from combined studies in the cBioPortal database. The graphical representation depicts the protein domain structure and specific mutation sites. The length of the lines connecting mutations to the protein reflects the number of samples exhibiting those mutations, and the color of the spheres indicates the type of mutation. (Lower) The bar chart shows the mutation frequencies across the SH3, BH, nSH2, iSH2, and cSH2 domains of the *PIK3R1* gene, as derived from combined studies of various tissues in the cBioPortal database.

*PIK3R1* is located on human chromosome 5 and comprises five protein domains: the Src homology 3 (SH3) domain, the breakpoint cluster region homology (BH) domain, the N-terminal SH2 (nSH2) domain, the middle SH2 (iSH2) domain, and the C-terminal SH2 (cSH2) domain. Notably, cancer-associated mutations have been identified in all five domains 27. Hotspot mutations in *PIK3R1* are predominantly found within the iSH2 and SH2 domains (**Fig. 2**), supporting a significant role for p110α in stabilizing and inhibiting p85α-p110α heterodimers through these domains 28.

### 1. SH3 and BH domains

The SH3 and BH domains at the N-terminus of p85 can form homodimers and bind to PTEN 8, 29, 30. When p85α is devoid of p110α, it can homodimerize via two intermolecular interactions (SH3: proline-rich region; BH: BH) to selectively bind unphosphorylated activated PTEN 31. PTEN, a tumor suppressor protein, is often lost or mutated in up to 30% of human cancers 32-34. Acting as a phosphatase, PTEN dephosphorylates the D3 position of PIP3, counteracting the activation of the oncogenic PI3K/AKT/mTOR (PAM) signaling network 35. The p85α-PTEN interaction is associated with enhanced PTEN stability by inhibiting ubiquitination 30. Furthermore, homodimers may offer a combinatorial binding site for PTEN and potentially promote the recruitment of other molecules to stabilize PTEN 30. Mutations in the SH3-BH domain diminish homodimerization and PTEN binding, leading to increased PTEN ubiquitination and reduced total PTEN protein levels. The p85α homodimer has been shown to compete with the E3 ubiquitin-protein ligase WWP2 for binding to the PTEN phosphatase domain, thus protecting PTEN from WWP2-mediated degradation. Disruption of p85α homodimerization increases WWP2-mediated PTEN degradation and enhances its ubiquitination 31. Notably, cellular expression of p85α lacking the SH3-BH domain significantly elevates the amplitude and duration of AKT phosphorylation in response to growth factor stimulation.

Additionally, the BH domain of the p85α protein exhibits sequence homology with the GAP domain of other proteins and displays GAP activity against several Rab GTPases, particularly Rab5 and Rab4. These GTPases play a crucial role in receptor trafficking and degradation, thus deactivating upstream receptor signaling and the PI3K/AKT pathway 3, 36. A study revealed that a RabGAP-deficient p85α mutant with a single-point mutation (R274A) has carcinogenic properties 37. Mutations in the p85α BH domain may contribute to tumorigenesis in human cancers, either by modulating Rab-mediated receptor degradation or by diminishing the positive regulation of PTEN activity 8. Consequently, mutations in the SH3-BH domain compromise PTEN stability by impairing homodimerization and PTEN binding, resulting in reduced negative feedback to the PI3K/AKT pathway and leading to persistent activation of downstream signals.

### 2. nSH2 domain

Driver mutations in the p85α nSH2 domain exert a precancerous effect by regulating the PI3Kα pathway through upstream signaling proteins. The p85α nSH2 domain establishes inhibitory contacts with p110α, competing for binding to the phosphorylated tyrosine-containing (pY) consensus sequences (pYXXM) motif in receptor tyrosine kinases (RTKs) 28, 38. The nSH2 domain interacts reversibly with the C2, helix, and kinase domains of p110α, with these contacts being disrupted upon binding to the pYXXM motif 39-41. Alkaline residues surrounding the phosphopeptide binding site in the nSH2 domain form inhibitory contacts with the acidic patch in the p110α helix domain. Binding of phosphoproteins to the nSH2 domain may disrupt these inhibitory contacts and activate the p85α/p110α dimer 39. The release of p85α's inhibitory effect on the p110α catalytic subunit occurs when the nSH2 domain binds to phosphorylated RTKs or adaptor proteins following upstream stimulation 42. Phosphopeptide binding to nSH2 directly influences the activity of p110α, establishing the nSH2 domain as a direct regulator of p110α 28.

Research has identified six nSH2 variants that attenuate these inhibitory contacts by directly affecting p110 binding (G376R, K379E, and L380del), or by disrupting the folding of the nSH2 domain (W333R, G353R, LR372del) 43. Furthermore, these driver mutations in the nSH2 domain of p85α activate various RTK signaling proteins, such as EGFR, HER2, HER3, c-Met, and IGF-1R, in a p110-independent manner, thereby diminishing the inhibitory effect of p85α on p110α and facilitating its catalytic activity 43. Mutations that disrupt the nSH2-helix interface were also found to weaken the interaction between the C2-iSH2 domains and the remainder of the adaptor and catalytic subunits, suggesting that the nSH2 domain not only inhibits enzyme activity via the C-leaf interaction with the kinase domain but also plays a crucial scaffold role in stabilizing the enzyme and preventing the inter-domain movements necessary for membrane binding 40, 44. Moreover, p85α mutants lacking the p110α binding region (e.g., R162*, L380fs, R348*, and the dominant negative mutant p85Δ) failed to interact with p110α 45. Notably, *PIK3R1*^R348*^ and *PIK3R1*^L370fs^ localize to the nucleus, serving as scaffolds for multiple JNK pathway components, and promote malignant phenotypes through ERK and JNK signaling pathways both *in vitro* and *in vivo* 46. Collectively, mutations in the nSH2 domain of p85α not only reduce the inhibitory interaction between p85α and p110α but also enhance p110α activity by inducing the activation of multiple RTKs.

### 3. iSH2 domain

Somatic mutations in *PIK3R1* have been identified in glioblastoma and endometrial cancer, predominantly manifesting as substitutions or indels within the iSH2 domain of p85α, which interacts with p110α 28, 30. These mutations lead to the uncoupling of p85α from p110, allowing p85α to retain its p110-stabilizing activity while abolishing its inhibitory effect on p110α. This alteration promotes cell survival, activates AKT, supports anchorage-independent cell growth, and facilitates tumorigenesis in a p110α-dependent manner 45. Furthermore, the iSH2 domain of p85α also engages catalytic subunits through the ABD domain. The ABD domain of class IA PI3K irreversibly binds to the coiled-coil domain of iSH2 across all class IA regulatory subunits 39, 47, 48. Because the ABD domain remains tightly associated with the iSH2 domain, its detachment may coincide with the disruption of the C2-iSH2 interface, with complete disruption of both interfaces only occurring upon removal of the nSH2 domain 48. These activating mutations within the iSH2 domain induce the constitutive activation of class IA PI3K kinases and contribute to tumorigenesis by disrupting the boundary between the C2 and iSH2 domains, diminishing the p110α-p85α interaction, and relieving the inhibition of PI3K activity *in vivo* 47, 49. Additionally, the oncogenic mutation from Glu545 to Lys545 (E545K) disrupts the charge-charge interaction with the nSH2 domain of p85 39. Consequently, driver mutations in the iSH2 domain diminish the inhibitory effect of p85α on p110 kinase activity by disrupting the inhibitory interactions between the iSH2 and p110 C2 domains and targeting the inhibitory interactions between the nSH2 domain and the p110 helix domain.

### 4. cSH2 domains

In addition to binding to p110, both the nSH2 and cSH2 domains interact with phosphorylated RTKs or pYXXM in adaptor proteins 28. The cSH2 domain of p85α serves as a negative regulatory element in PI3K signaling, with its deletion resulting in increased signaling activity 50. Notably, mutations in the cSH2 domain, including the oncogenic truncation mutation (E601*) and the mutation affecting the phosphorylated peptide binding site (R649W) in SHORT syndrome, both lead to diminished sensitivity to PDGFR pY, underscoring the essential role of cSH2 in mediating effective PI3K signaling downstream of activated membrane receptors 51, 52. Furthermore, the impact of truncated mutants lacking the cSH2 domain on PI3K signaling may mimic a decrease in p85α levels, potentially facilitating engineered cellular responses by increasing available binding sites on activated RTKs, which may promote the formation of signaling-competent p110-p85α heterodimers 14. An oncogenic variant termed p65-PI3K, which lacks the cSH2 domain and possesses the capacity for constitutive activation and cell transformation, has been isolated from irradiation-induced mouse thymic lymphoma. Additionally, a C-terminal truncated version of p85α, known as p76α, has been identified in human lymphoma cell lines, lacking substantial portions of the cSH2 domain due to frameshift mutations 53-55. While the cSH2 domain of p85α does not establish an inhibitory interface with the catalytic subunit, its presence may be essential for the complete inhibition of catalytic subunit activity 51, 56. Deletion of the cSH2 domain can augment the signaling activity of the nSH2 mutant of p85α, and mutations in the cSH2 domain can abolish its negative regulatory influence on PI3K activity through the nSH2 domain of p85α 50, 55. In summary, mutations in the cSH2 domain can diminish the negative regulatory function of p85α on PI3K activity, thereby modulating the catalytic activity of PI3K.

### 5. PI3K mutations in HPV-associated tumors

Human papillomavirus (HPV) is a well-established oncogenic virus. Studies have demonstrated that infection with high-risk HPV types is a major risk factor for the development of cervical, vaginal, and vulvar cancers 57. Real-world data indicate that 48% of HPV-associated gynecological tumors harbor actionable mutations, with PI3K mutations being among the most prevalent oncogenic events 57. Additionally, approximately 25% of oropharyngeal squamous cell carcinoma (OPSCC) cases worldwide are also attributed to HPV infection 58. Compared to HPV-negative OPSCC patients, those with HPV-positive OPSCC demonstrate significantly improved overall survival and heightened sensitivity to chemotherapy, radiotherapy, and combined chemoradiotherapy 59-61. Furthermore, HPV-positive OPSCC exhibits a distinct mutational landscape compared to its HPV-negative counterpart 62. Among HPV-positive OPSCC cases, the class I subgroup of the PI3K family is most frequently associated with mutational dysregulation. Notably, the PI3K/PTEN/AKT/mTOR pathway has been identified as a critical oncogenic driver in HPV-positive cohorts 58. Epidemiological studies indicate that 13% to 25% of HPV-positive OPSCC patients experience local or distant recurrence (LDR), which is associated with significantly reduced survival rates 59. HPV-positive OPSCC patients who develop LDR exhibit a higher mutation burden compared to those without LDR, with mutation frequencies comparable to those observed in HPV-negative OPSCC patients with LDR 59. Interestingly, HPV-negative OPSCC patients without LDR exhibit the highest overall mutation burden, suggesting that recurrence may be driven more by specific oncogenic mutations rather than total mutation load 59. In HPV-positive OPSCC, *PIK3R1* mutations occur at a higher frequency in LDR patients than in non-LDR patients 59. Evidence suggests that aberrant PI3K signaling may contribute to cancer progression and poor prognosis by altering *PIK3R1* and destabilizing the *PIK3CA/PIK3R1* complex 63.

## Epigenetic Alterations Occurring in the *PIK3R1* Gene

Accumulating evidence indicates that aberrant epigenetic regulation of gene function is closely linked to the development of cancer 64. Cell transformation, tumor progression, and metastasis are orchestrated by a complex network of interactions involving genomic and epigenomic mutations, particularly those affecting oncogenes and tumor suppressor genes, along with environmental factors that contribute to malignancy and tumorigenesis 65, 66.

### 1. DNA methylation

Higher levels of promoter methylation of *PIK3R1* have been observed across various cancer types, and this methylation is positively correlated with gene expression levels in multiple probes within the promoter region 11. Notably, hypomethylation of the CpG locus in the *PIK3R1* promoter is associated with reduced gene expression and correlates with decreased overall survival and relapse-free survival in pancreatic cancer patients 67. Furthermore, the potential of *PIK3R1* methylation as a biomarker has been reported in esophageal cancer 68. In breast cancer, the downregulation and hypermethylation of *PIK3R1* correlate with poor patient outcomes, suggesting its utility as a diagnostic and prognostic biomarker for breast cancer 69. Consequently, the methylation level of *PIK3R1* is positively correlated with expression levels and is closely related to clinical data from cancer patients, establishing it as a promising cancer biomarker.

### 2. Histone modifications

The isonicotinylation of lysine residues on histones diminishes the binding capacity between histones and genomic DNA, resulting in a more open chromatin structure that facilitates the transcription of numerous genes 70, 71. In isoniazid-treated HepG2 cells, RNA sequencing analysis has revealed an upregulation of the *PIK3R1* gene, mediated by isoniazid through histone modifications, leading to elevated levels of p85α and the activation of the hepatocellular carcinoma-associated PAM pathway 70. There exists a CapG-binding region within the *PIK3R1* gene, situated near the transcription start site of *PIK3R1* variant 3 (P50) 72. The transcriptional coactivator CREB-binding protein/p300 is recruited to the *PIK3R1* promoter via interactions with CapG, enhancing the transcription of *PIK3R1/P50* through histone H3 lysine 27 acetylation (H3K27Ac) 72. This process triggers the activation of the PI3K/AKT signaling pathway, contributing to paclitaxel resistance in breast cancer cells. Chemoresistance in hepatocellular carcinoma is partially attributed to chemotherapy-induced N-acetyltransferase 10, which significantly upregulates H3K27Ac on the *PIK3R1* promoter, thereby activating transcription and driving chemoresistance 73. Additionally, a super-enhancer characterized by a high level of H3K27Ac and mediator bindings has been identified at the *PIK3R1* locus in multiple adult T-cell leukemia/lymphoma samples, but not in normal T cells 74. This finding suggests an involvement of the PI3K/AKT pathway in the pathogenesis of adult T-cell leukemia/lymphoma 74. Overall, histone modifications of *PIK3R1* may enhance p85α expression, thus activating the PI3K/AKT pathway and potentially contributing to tumor chemoresistance.

### 3. Non-coding RNA

MicroRNAs (miRNAs) are endogenous non-coding RNAs approximately 21 nucleotides in length, which are expressed in most somatic tissues 75. They regulate gene expression post-transcriptionally and are integral components of the epigenome. In many cancers, miRNA expression is dysregulated, with *PIK3R1* being targeted by multiple miRNAs that exert tumor-suppressive effects 76, 77. For instance, *PIK3R1* has been identified as a direct target of miR-155 in breast cancer and B lymphocytes, where it promotes tumor growth by activating glucose metabolism 78-80. In ovarian cancer, circPLPP4 targets miR-136, acting as a competitive endogenous RNA to regulate *PIK3R1* expression and enhance cisplatin resistance 81. Additionally, p85α, a crucial target of miR-29 in the p53 pathway, upregulates p53, inducing apoptosis in a p53-dependent manner through a PI3K/AKT/MDM2-mediated mechanism 82. Furthermore, p85α is a direct target of miR-503; its ectopic expression inhibits tumor cell proliferation and metastasis-related traits both *in vitro* and *in vivo* 83, 84. The modulation of miR-503 through overexpression and knockdown partially influences apoptotic activity and alters cisplatin resistance in ovarian cancer cells, suggesting that miR-503 may serve as a sensitizer for cisplatin therapy in ovarian cancer 85. In colorectal cancer, miR-455-5p acts as a tumor suppressor by inhibiting the proliferation and migration of colorectal cancer cells while promoting their apoptosis; it may also target and downregulate *PIK3R1*, increasing sensitivity to 5-fluorouracil 86, 87. Moreover, miR-21's targeting of *PIK3R1* inhibits tumor cell migration and invasion by reducing PI3K/AKT signaling, reversing epithelial-to-mesenchymal transition, and predicting clinical outcomes in breast cancer 15. miR-495 similarly promotes endometrial cell apoptosis and inhibits proliferation by also targeting *PIK3R1* 88. Overall, *PIK3R1* emerges as a target of multiple miRNAs and plays a significant role in the initiation and progression of various cancers.

Circular RNAs (circRNAs) are single-stranded, covalently closed RNA molecules formed by the reverse splicing of mRNA exons 89. Many circRNAs exhibit aberrant expression across various cancers, with their dysregulation being closely associated with tumor progression 90. In hepatocellular carcinoma cells, circRHBDD1 acts as a scaffold, enhancing the interaction between YTHDF1 and *PIK3R1* mRNA and promoting *PIK3R1* mRNA translation in an m6A-dependent manner 80. CircSEMA4B, a protein-coding circRNA significantly downregulated in ovarian cancer, encodes a novel protein, SEMA4B-211aa, which inhibits PIP3 production by binding to p85, thereby inhibiting AKT phosphorylation and breast cancer progression 91. CircRNAs can exert either anti-immunotherapeutic or anti-tumor effects by regulating the expression of *PIK3R1* or binding to p85α.

## Clinical Drugs Targeting the PAM Pathway and Targeted Therapies for Cancers Harboring *PIK3R1* Aberrations

### 1. FDA-Approved Clinical Drugs Targeting the PAM Pathway

Due to the alteration of the PI3K pathway being found in multiple cancers, it is considered one of the most commonly targeted signaling cascades, and as a result, several PI3K/AKT inhibitors have been developed 57, 92. Several classes of drugs target the PI3K pathway, including pan-PI3K inhibitors, isoform-selective PI3K inhibitors (IS PI3Ki), AKT inhibitors, mTOR inhibitors, and dual PI3K/mTOR inhibitors 93. Pan-PI3K inhibitors effectively target high-level PIP3 tumors by inhibiting the catalytic activity of all four class I PI3K isoforms: PI3Kα, PI3Kβ, PI3Kγ, and PI3Kδ 94. While numerous pan-PI3K inhibitors are currently in clinical development, copanlisib is the only one to have demonstrated significant efficacy in clinical trials 95. IS PI3Ki specifically inhibit selected PI3K isoforms (α/β/γ or δ), and of the IS PI3Ki, only six have received Food and Drug Administration (FDA) approval: alpelisib, umbralisib, duvelisib, inavolisib, leniolisib, and idelalisib 94, 96-102. Alpelisib, a potent drug with targeted efficacy against the PI3Kα isoform, has been approved for use in patients with advanced or metastatic breast cancer with hormone receptor (HR)+/HER2-*PIK3CA* mutations in combination with fulvestrant 94.

AKT inhibitors are classified into three broad categories: ATP-competitive, allosteric, and covalent allosteric inhibitors, with capivasertib being the first drug to receive FDA approval in combination with the estrogen receptor degrader fulvestrant for breast cancer treatment 103.

mTOR inhibitors were the first PAM-targeted drugs to advance to clinical use 104, 105. These inhibitors are generally classified into three generations based on their common binding sites on mTOR. Current clinical options—rapamycin, temsirolimus, everolimus, and rapamycin bound to albumin—belong to the first generation 106. The FDA-approved mTOR inhibitors are everolimus, sirolimus, and temsirolimus. Dual PI3K/mTOR inhibitors target both PI3K and mTOR signaling; however, none are currently FDA-approved for cancer treatment 96. PI3K/mTOR dual ATP-competitive inhibitors directly act on PI3K and mTOR, more effectively inhibiting the PAM signaling pathway, and reducing resistance and side effects compared to single inhibitors 107-109. Reports of dual PI3K/mTOR inhibitors (Gedatolisib, Omipalisib, Apitolisib, and others) currently in Phase I or II clinical stages indicate that none have received FDA approval for cancer therapy 96, 110-113.

Normal cells depend on PI3K signaling for survival; consequently, serious adverse effects may present before complete inhibition of target tumor cells 114. Over 40 PI3K pathway inhibitors are undergoing various clinical development stages 114, yet few have received FDA approval (**Table 1**). Most PI3K inhibitors provide only modest benefits, thus limiting their application in clinical settings.

Understanding the aberrant expression of cancer pathway genes that play crucial roles in cancer initiation and progression can positively impact clinical outcomes for patients with genetic anomalies. The degree to which these aberrations drive tumor behavior and serve as critical therapeutic targets necessitates further investigation to establish predictive biomarkers of response to PI3K inhibition 115.

### 2. Targeted therapy for *PIK3R1* gene aberrations in pre-clinical studies

Specific molecular aberrations in cancer-associated genes may have functional consequences that influence treatment sensitivity 46, 116, 117. Selective pharmacological inhibition of Pan-PI3K and p110α effectively block transformations driven by partial p85α deletion in both *in vitro* and *in vivo* models, indicating that p85α functions as a tumor suppressor in transformation processes. This suggests that p110α may represent a potential therapeutic target for treating breast cancer patients with *PIK3R1* deletions 14. The blockade of AKT or STAT3 may benefit ovarian cancer patients experiencing copy number losses or reduced expression of *PIK3R1*. The combination of AKT and STAT3 inhibitors demonstrates synergistic antitumor effects *in vitro* in 3D spheroid models and shows enhanced efficacy *in vivo* compared to either inhibitor alone 20.

*PIK3R1* mutations across different domains exhibit varying impacts on cancer, necessitating diverse therapeutic strategies for effectiveness 3. A growing body of evidence suggests that the efficacy of these drugs may partly depend on specific mutations in target proteins and the genetic context surrounding those mutations. A comprehensive understanding of how *PIK3R1* mutations affect their interactions and regulatory functions will aid in identifying cancer-associated mutations that dysregulate the PI3K pathway and pinpoint the most effective therapeutic targets for inhibitor therapy 3. While a small percentage of patients have observed clinical benefits from treatments based on newly identified hotspot mutations, further exploration into the ramifications of various combinations of mutations and genetic alterations on cancer biology and therapeutic sensitivity complicates the landscape 118.

Mutation characteristics in cancer cells provide insights into tumorigenesis and reveal candidates for targeted therapies. Different mutations can result in varying susceptibilities to specific PI3K pathway inhibitors 30. Inhibitors targeting the AKT or HER families can diminish the carcinogenic potential of driver mutations, with combined use yielding significant synergistic effects 43. Cells harboring nSH2 domain-driven mutations exhibit similar traits to those expressing HER3 43. Notably, HER3-expressing cells activate the PI3K pathway without engaging the MAPK pathway 119, 120. In 19 endometrial cancer cell lines, HER3 overexpression correlated with responsiveness to the dual epidermal growth factor receptor-1/2 inhibitor lapatinib 121. Furthermore, patients with *PIK3R1*^R348*^, *PIK3R1*^L370fs^, or adjacent *PIK3R1* mutations may benefit from concurrent treatment with RAS and PI3K pathway inhibitors, conferring unexpected sensitivity to MEK and JNK inhibitors in both *in vitro* and *in vivo* settings 46. Targeting p85α homodimerization or p85α:PTEN interactions may present new therapeutic avenues for mutations in the p85α BH and SH3 domains 31. Mutations in the iSH2 domain may facilitate the development of pharmacological compounds that inhibit PI3-kinase by stabilizing the regulated C2-iSH2 interface between p85 and p110 47. Tumors exhibiting iSH2 mutations are likely to respond favorably to inhibitor therapies targeting PI3K or its downstream effectors, such as AKT 45.

Given the significance of epigenetic alterations in cancer and their potential for reprogramming, molecular regulators of these modifications have emerged as promising targets in cancer therapy 64, 122. Currently, the most extensively studied epigenetic molecular inhibitors include histone deacetylase inhibitors, histone lysine demethylase inhibitors, and DNA methyltransferase inhibitors, alongside dietary interventions involving epigenetically modified proteins and metabolic molecules as promising treatment strategies 123. However, responses to monotherapy often fall short of expectations, with resistance to such therapies appearing inevitable 124. The introduction of new therapies, including the application of miRNAs, multidrug combinations, and immunotherapies, may enhance cancer treatment outcomes while mitigating drug resistance in comparison to monotherapy 64, 125-127. Various combination strategies have been proposed and tested, necessitating further research to bolster the potential of these epigenetic drugs as innovative treatments.

## Conclusion

We summarize evidence indicating that *PIK3R1* manifests characteristics consistent with a tumor suppressor gene. In several cancer types, *PIK3R1* experiences copy number loss, leading to the activation of downstream signaling molecules that promote cancer development, suggesting that the occurrence of *PIK3R1* copy number deletion may be a key indicator linked to poor cancer prognosis. Cancer-associated mutations have been identified across all five protein domains of the *PIK3R1* gene. Driver mutations in the BH and SH3 domains can diminish the negative feedback of the PI3K/AKT pathway by reducing homodimerization or binding to PTEN, thereby activating downstream signals. Driver mutations within the iSH2 domain can trigger the downstream AKT signaling pathway by weakening the interaction between p110α and p85α. Meanwhile, driver mutations found in the nSH2 domain may exert catalytic effects by activating a series of upstream RTK signaling proteins to diminish the inhibitory effect of p85α on p110α. However, there is a scarcity of comprehensive literature regarding mutations in the cSH2 domain, necessitating further investigations to elucidate the oncogenic mechanisms underlying driver mutations in this domain. Additionally, epigenetic changes associated with the *PIK3R1* gene remain a focal point of research, and *PIK3R1* methylation could potentially serve as a cancer biomarker indicative of malignancy prognosis, as well as a target for multiple miRNAs that perform tumor-suppressive functions, thus providing a robust foundation for future development of specific epigenetic drugs.

In conclusion, *PIK3R1* plays a crucial role in cancer as a regulatory subunit of PI3K, and the phenotypic variations observed in human cancers stem from a complex interplay of genetic factors, transcriptomic profiles, epigenetics, and proteomics. Copy number variations, mutations, and epigenetic alterations in *PIK3R1* contribute to a proliferative phenotype characterized by recurrent mutations across diverse cancers. Developing therapeutic strategies that target the PI3K signaling network holds significant promise for enhancing cancer treatment outcomes. The accumulating body of evidence related to genetic aberrations in *PIK3R1* and corresponding cancer markers reinforces the notion that targeting the aberrant *PIK3R1* pathway could be advantageous for anticancer therapy.

## Figures and Tables

**Table 1:** FDA-approved inhibitors of the PAM pathway

Drug name	Target	Indication	Launch year
Umbralisib	PI3Kδ; CK1ε	Adults with relapsed or refractory marginal zone and follicular lymphoma.	2021
Alpelisib	PI3Kα	Alpelisib in combination with fulvestrant for postmenopausal women, and men, with hormone receptor (HR)-positive, human epidermal growth factor receptor 2 (HER2)-negative, *PIK3CA*-mutated, advanced or metastatic breast cancer.	2019
Duvelisib	PI3Kδ; PI3Kγ	Chronic lymphocytic leukemia/small lymphocytic lymphoma and follicular lymphoma.	2018
Copanlisib	Pan-class PI3K	Adults with relapsed follicular lymphoma who have received at least two prior systemic therapies.	2017
Idelalisib	PI3Kδ	Relapsed/refractory chronic lymphocytic leukemia, follicular lymphoma, and small lymphocytic lymphoma.	2014
Inavolisib	PI3Kα	Inavolisib with palbociclib and fulvestrant for endocrine-resistant, *PIK3CA*-mutated, HR-positive, HER2-negative, advanced breast cancer.	2024
Leniolisib	PI3Kδ	Adults and children 12 years of age or older with Activated Phosphoinositide-3-kinase-delta Syndrome (APDS).	2023
Capivasertib	Pan-AKT	Capivasertib with fulvestrant for adult patients with hormone receptor (HR)-positive, human epidermal growth factor receptor 2 (HER2)-negative locally advanced or metastatic breast cancer with one or more *PIK3CA/AKT1/PTEN*-alterations.	2023
Everolimus	mTOR	Postmenopausal women with advanced hormone receptor-positive, HER2-negative breast cancer in combination with exemestane, after the failure of treatment with letrozole or anastrozole. Adult patients with progressive neuroendocrine tumors of pancreatic origin (PNET) with unresectable, locally advanced or metastatic disease. Adult patients with advanced RCC after failure of treatment with sunitinib or sorafenib. Adult patients with renal angiomyolipoma and tuberous sclerosis complex (TSC), not requiring immediate surgery. Adult and pediatric patients, 3 years of age or older, with SEGA associated with TSC who require therapeutic intervention but are not candidates for curative surgical resection.	2009
Sirolimus	mTOR	The prophylaxis of organ rejection in patients aged ≥13 years receiving renal transplants.	1999
Temsirolimus	mTOR	Advanced renal cell carcinoma.	2007

## Abbreviations

PI3K: phosphoinositide 3-kinase
PIP3: phosphatidylinositol 3,4,5-triphosphate
TCGA: The Cancer Genome Atlas
SH3: Src homology 3
BH: breakpoint cluster region homology
nSH2: N-terminal SH2
iSH2: middle SH2
cSH2: C-terminal SH2
PAM: PI3K/AKT/mTOR
pY: phosphorylated tyrosine-containing
pYXXM: phosphorylated tyrosine-containing consensus sequences
RTKs: receptor tyrosine kinases
HPV: Human papillomavirus
OPSCC: oropharyngeal squamous cell carcinoma
LDR: local or distant recurrence
H3K27Ac: histone H3 lysine 27 acetylation
miRNAS: MicroRNAs
circRNAs: Circular RNAs
IS PI3Ki: isoform-selective PI3K inhibitors
FDA: Food and Drug Administration
